# Dermal mesenchymal stem cells promote angiogenesis in HMEC-1 via activation of the angiopoietin 1/Tie2 pathway in psoriasis

**DOI:** 10.3389/fcell.2026.1771279

**Published:** 2026-05-20

**Authors:** Jiao Li, Hui Hou, Jianxiao Xing, Junqin Li, Kaiming Zhang

**Affiliations:** Shanxi Key Laboratory of Stem Cells for Immunological Dermatosis, Institute of Dermatology, Taiyuan Central Hospital, Taiyuan, Shanxi, China

**Keywords:** angiogenesis, angiopoietin 1, dermal mesenchymal stem cells, psoriasis, Tie2

## Abstract

**Background:**

Abnormal proliferation of microvessels in the superficial dermis is a key pathological feature of psoriasis. The angiopoietin 1 (Ang1)/Tie2 axis regulates angiogenesis and is closely associated with the cellular microenvironment. Our previous studies demonstrated that dermal mesenchymal stem cells (DMSCs) derived from psoriatic lesions can promote angiogenesis in human umbilical vein endothelial cells; however, the link between this proangiogenic effect and the Ang1/Tie2 axis remains unclear.

**Objective:**

To investigate the potential role of Ang1 in DMSC-mediated promotion of angiogenesis in human dermal microvascular endothelial cells (HMEC-1) in the context of psoriasis.

**Methods:**

DMSCs were isolated from the skin of 18 psoriatic patients and 18 healthy individuals. Normal DMSCs (N-DMSCs) with ANGPT1 overexpression and psoriatic DMSCs (P-DMSCs) with ANGPT1 knockdown were co-cultured with HMEC-1, respectively. qRT-PCR and Western blotting were used to detect the expression levels of Ang1, Tie2, and other related genes. CCK-8, EdU staining, Transwell chamber, and Matrigel tube formation assays were employed to evaluate HMEC-1 function. A psoriasis mouse model was established using IMQ, and HE staining was performed to measure the epidermal thickness of lesional skin.

**Results:**

Compared with N-DMSCs, P-DMSCs exhibited significantly higher mRNA and protein expression levels of Ang1. Co-culture of HMEC-1 with ANGPT1-overexpressing N-DMSCs increased the phosphorylation level of Tie2 in HMEC-1, along with enhanced cell proliferation, migration, tube formation, and upregulated expression of angiogenesis-related genes. These proangiogenic effects were attenuated following the addition of a PI3K inhibitor. In contrast, co-culture with ANGPT1-knockdown P-DMSCs inhibited HMEC-1 function, and this inhibitory effect could be reversed by treatment with a PI3K activator. Additionally, ANGPT1 overexpression exacerbated psoriatic symptoms in IMQ-induced mice, while ANGPT1 knockdown alleviated these symptoms.

**Conclusion:**

P-DMSCs promote the proliferation, migration, and tube formation of HMEC-1 by upregulating Ang1, which activates Tie2 and the downstream PI3K/AKT pathway. These findings provide a theoretical basis for understanding the mechanism of abnormal angiogenesis in psoriasis and support the development of targeted therapies for this disease.

## Introduction

Psoriasis is a common chronic inflammatory skin disorder characterized by three core pathological features: excessive keratinocyte proliferation, inflammatory cell infiltration, and abnormal proliferation of microvessels in the superficial dermis (Afonina et al., 2021). Among these, abnormal angiogenesis is recognized as a critical driver of the formation and maintenance of psoriatic lesions. Overproliferated blood vessels not only supply nutrients to lesional tissues but also exacerbate local inflammatory responses and tissue remodeling through crosstalk with inflammatory cells and keratinocytes, thereby sustaining and amplifying the pathological microenvironment of the lesions (Zhang et al., 2025). Current studies indicate that an imbalance between proangiogenic and antiangiogenic factors in psoriatic lesions contributes to abnormal angiogenesis (Socha et al., 2021); however, the specific regulatory mechanisms underlying this imbalance remain to be fully elucidated.

Dermal mesenchymal stem cells (DMSCs) are a key population of stem cells in skin tissue, endowed with multipotent differentiation potential and immunomodulatory capabilities. They play essential roles in skin tissue repair, regeneration, and the regulation of inflammatory responses (Xia et al., 2025). Recent studies have confirmed that DMSCs can secrete a variety of cytokines and growth factors via paracrine signaling, participating in physiological and pathological processes such as skin tissue repair, immune regulation, and vascular regeneration (Niu et al., 2019; Liu et al., 2020; Liu et al., 2022). Our previous research showed that, compared with normal DMSCs, DMSCs derived from psoriatic lesions exhibit enhanced *in vitro* differentiation into vascular endothelial cells (ECs) (Li et al., 2018), accompanied by abnormal gene expression (Hou et al., 2014), dysregulated cytokine secretion (Liu et al., 2013), impaired inhibition of activated T-cell proliferation (Liu et al., 2014), abnormal methylation of gene promoters (Hou et al., 2013), and glucose metabolism disorders (Zhao et al., 2021). Notably, angiogenesis-related genes (e.g., Neuropilin 2, Vasohibin-2, and transcription factor GATA-6) are also dysregulated in these cells (Niu et al., 2016a; Niu et al., 2016b). Furthermore, DMSCs from psoriatic lesions can induce angiogenesis in human umbilical vein endothelial cells *in vitro* (Zhou et al., 2021). Nevertheless, the effect of DMSCs on the angiogenic capacity of human dermal microvascular endothelial cells (HMEC-1) and the underlying mechanisms remain unclear.

The signaling pathway composed of angiopoietin 1 (Ang1) and its receptor Tie2 (Tyrosine kinase with immunoglobulin-like and EGF-like domains 2) plays a central role in maintaining vascular stability, promoting vascular maturation, and facilitating angiogenesis (Zhou et al., 2022). Ang1, primarily secreted by stromal cells, binds specifically to the Tie2 receptor on the surface of ECs, activating downstream signaling pathways such as PI3K/AKT and ERK. This activation regulates EC proliferation, migration, adhesion, and tube formation, representing a key molecular mechanism for maintaining normal vascular structure and function (Li et al., 2025). Previous studies have confirmed that abnormal activation or inhibition of the Ang1/Tie2 pathway is closely associated with various vascular proliferative diseases (Joussen et al., 2021), and dysregulated expression of molecules in this pathway has been detected in psoriatic lesions (Kuroda et al., 2001), suggesting that the Ang1/Tie2 axis may be involved in the regulation of vascular pathological changes in psoriasis. However, the specific regulatory mechanism of the Ang1/Tie2 pathway in psoriasis-associated angiogenesis requires further investigation.

This study aimed to explore the role and mechanism of DMSCs in promoting HMEC-1 angiogenesis via activation of the Ang1/Tie2 pathway in psoriasis. The findings provide new experimental evidence for clarifying the molecular mechanisms of abnormal vascular proliferation in psoriasis and lay a theoretical foundation for the development of therapeutic strategies targeting DMSCs or the Ang1/Tie2 pathway.

## Materials and methods

### Patient enrollment

18 patients with plaque psoriasis who attended the Department of Dermatology at Taiyuan Central Hospital from January to December 2023 were enrolled in this study. The cohort included 10 males and 8 females, aged 13–60 years (mean age: 36.72 ± 2.80 years), with disease durations ranging from 3 months to 10 years (mean duration: 48.18 ± 7.85 months). 18 age- and gender-matched healthy individuals without a history of psoriasis were selected as controls. None of the participants had used immunosuppressants, corticosteroids, or retinoids within 3 months prior to sample collection. This study was approved by the Ethics Committee of Taiyuan Central Hospital and conducted in accordance with the principles of the Declaration of Helsinki. Written informed consent was obtained from all participants before the study.

### Isolation, culture, and identification of DMSCs

Primary DMSCs from psoriatic patients and healthy individuals were isolated and cultured as previously described (Hou et al., 2014). Skin tissue was cut into ∼1 mm^3^ fragments, which were then digested with 0.25% Dispase II in DMEM/F12 medium at 37 °C for 2–4 h to separate the dermis from the epidermis. The dermis was collected, minced, and homogenized by repeated pipetting. Undigested tissue debris was removed using a 40 μm cell strainer, and the filtrate was centrifuged. The resulting cell pellet was resuspended in DMEM/F12 medium supplemented with 10% fetal bovine serum (FBS) and inoculated into T25 culture flasks. Cells were cultured at 37 °C in a humidified incubator with 5% CO_2_. Cell morphology was observed and recorded using an inverted phase-contrast microscope. When cells reached 90% confluence, they were digested with 0.25% trypsin for passage. Passage 3 cells were collected, washed with PBS, and counted. Flow cytometry was used to identify the immunophenotype of DMSCs (CD29, CD44, CD34, CD105, CD45, HLA-DR). Adipogenic and osteogenic induction media were used to induce *in vitro* differentiation of the collected cells (Hou et al., 2014). For cytokine stimulation experiments, Normal DMSCs (N-DMSCs) at passages 3-5 were seeded into 6-well plates at a density of 2 × 10^5^ cells per well and cultured overnight. Cells were then treated with IL-17A (10 or 50 ng/mL) and/or TNF-α (10 or 50 ng/mL) (both from PeproTech, USA) for 48 h. Finally, N-DMSCs were harvested for subsequent experiments.

### ANGPT1 plasmid construction and transfection

To upregulate or knockdown ANGPT1 expression in DMSCs, plasmids encoding ANGPT1-overexpressing sequences (pLVX-ANGPT1^high^), short hairpin RNA targeting ANGPT1 (pLKO.1-shANGPT1), and their corresponding negative controls (pLVX-Vehicle, pLKO.1-shNC) were purchased from PPL Corporation (PPL, China). DMSCs in the logarithmic growth phase were seeded into 6-well plates. When cells reached 90% confluence, they were transfected with the aforementioned plasmids using Lipofectamine 3000 transfection reagent (Invitrogen, USA) at 37 °C for 6 h, after which the medium was replaced. Cells were collected for subsequent experiments following 24 h of incubation.

### RNA isolation and quantitative real-time PCR (qRT-PCR)

Total RNA was extracted from cells using Trizol reagent (Invitrogen, USA) and purified via chloroform/isopropanol/ethanol precipitation. First-strand cDNA was synthesized using the PrimeScript RT Master Mix (Takara, Japan). qRT-PCR reactions were performed using TB Green Premix Ex Taq™ II (Takara, Japan) on an ABI QuantStudio 3 Real-Time PCR System (Applied Biosystems, USA). Relative gene expression was calculated using the 2^−ΔΔCt^ method, with β-actin serving as the endogenous reference gene for normalization. All experiments were performed in triplicate. The specific primer sequences for each gene are listed in Tables 1, 2.

**TABLE 1:** PCR primer sequences for DMSCs and HMEC-1.

Gene	Sequence (5′-3′)
β-actin	Forward: CTACAATGAGCTGCGTGTGGC Reverse: CAGGTCCAGACGCAGGATGGC
ANGPT1	Forward: TGCAGAGAGATGCTCCACAC Reverse: ATGGTAGCCGTGTGGTTCTG
TIE2	Forward: TTAGCCAGCTTAGTTCTCTGTGG Reverse: AGCATCAGATACAAGAGGTAGGG
Ki67	Forward: AGCACCTGCTTGTTTGGAAG Reverse: ATATTGCCTCCTGCTCATGG
CCND1	Forward: GTGCTGCGAAGTGGAAACC Reverse: ATCCAGGTGGCGACGATCT
CDC42	Forward: CGACCGCTGAGTTATCCACA Reverse: TTGACAGCCTTCAGGTCACG
RAC1	Forward: AGGCCATCAAGTGTGTGGTG Reverse: AAGAACACATCTGTTTGCGGA
VEGFA	Forward: ATCGAGTACATCTTCAAGCCAT Reverse: GTGAGGTTTGATCCGCATAATC
ICAM1	Forward: TCTTCCTCGGCCTTCCCATA Reverse: AGGTACCATGGCCCCAAATG
VCAM1	Forward: GGACCACATCTACGCTGACA Reverse: TTGACTGTGATCGGCTTCCC
ANGPT2	Forward: GGGACAGCCGGCAAAATAAG Reverse: CAAACCACCAGCCTCCTGTT
DLL4	Forward: TGAAAAGCCAGAGTGTCGGAT Reverse: CTCCTGCCTTATACCTCCGT
JAG1	Forward: CGGGAAGTGCAAGAGTCAGT Reverse: TTGGTTTCACAGTAGGCCCC
FGF1	Forward: GTGGATGGGACAAGGGACAG Reverse: ATTTGGTGTCTGTGAGCCGT
FGF2	Forward: GGGGTGGAGATGTAGAAGATGT Reverse: CTGGGGTTCACGGATGGG
PDGFA	Forward: CTGCCCATTCGGAGGAAGAG Reverse: AGATCAGGAAGTTGGCGGAC
PDGFB	Forward: CTCTTCCTGTCTCTCTGCTGCTAC Reverse: AGGAGCGGATCGAGTGGTC
CXCL8	Forward: CTCCAAACCTTTCCACCCCA Reverse: TTCTCAGCCCTCTTCAAAAACT
CXCL12	Forward: ATTCTCAACACTCCAAACTGTGC Reverse: ACTTTAGCTTCGGGTCAATGC
MMP2	Forward: ACAAAGAGTTGGCAGTGCAATA Reverse: TCTGGGGCAGTCCAAAGAAC
MMP9	Forward: CGGTTTGGAAACGCAGATGG Reverse: TGGGTGTAGAGTCTCTCGCT

**TABLE 2:** PCR primer sequences for mice.

Gene	Sequence (5′-3′)
mβ-actin	Forward: GATATCGCTGCGCTGGTCG Reverse: CATTCCCACCATCACACCCT
mIL-17A	Forward: GAGTAAAGCCCTACGCTCGAA Reverse: CTCCTCTTGTTGGACAACCAC
mIL-23	Forward: AATAATGTGCCCCGTATCCAGT Reverse: GCTCCCCTTTGAAGATGTCAG
mTNF-α	Forward: GATCGGTCCCCAAAGGGATG Reverse: TTTGCTACGACGTGGGCTAC
mVEGFA	Forward: CCCACGTCAGAGAGCAACAT Reverse: CCGGGATTTCTTGCGCTTTC
mPECAM-1	Forward: GGAAGTGTCCTCCCTTGAGC Reverse: GGGAGCCTTCCGTTCTTAGG
mKi67	Forward: ACCATCATTGACCGCTCCTTT Reverse: TTGACCTTCCCCATCAGGGT
mCDK2	Forward: GGCTCGACACTGAGACTGAA Reverse: GACACGGTGAGAATGGCAGA
mANGPT1	Forward: AGGCTTGGTTTCTCGTCAGA Reverse: TCCTCCCTTTAGCAAAACACCT

### Western blotting

Total proteins were extracted from cells and tissues using RIPA lysis buffer (Solarbio, China) supplemented with protease and phosphatase inhibitor cocktails (KeyGEN, China). Protein concentration was determined using a BCA Protein Assay Kit (Solarbio, China). Proteins were separated using the ProteinSimple Wes system (ProteinSimple, USA), an automated quantitative protein expression analysis platform for ultra-micro samples, according to the manufacturer’s instructions. Primary antibodies against Ang1, VEGFA, CD31, Neuropilin 1, Cyclin D1, Cdk2, PI3K, phosphorylated (p)-PI3K, AKT, p-AKT, STAT3, p-STAT3 and β-actin (all from Abcam, UK) and Tie2, p-Tie2, ERK1/2 and p-ERK1/2 (all from CST, USA) were used. HRP-conjugated goat anti-rabbit IgG (ProteinSimple, USA) was used as the secondary antibody. β-actin served as the loading control, and all protein levels were normalized to β-actin expression.

### Co-culture of DMSCs with HMEC-1 or primary HDMECs

HMEC-1 were purchased from Shanghai Zhong Qiao Xin Zhou Biotechnology (Zhong Qiao Xin Zhou, China). These immortalized cells were cultured in MCDB131 medium (Zhong Qiao Xin Zhou, China) supplemented with 10% FBS, 10 ng/mL EGF, 1 μg/mL hydrocortisone, 2 mM L-alanyl-L-glutamine, 100 U/mL penicillin, and 100 μg/mL streptomycin at 37 °C in a humidified incubator with 5% CO_2_. Primary human dermal microvascular endothelial cells (HDMECs) were isolated from skin tissues of healthy individuals as previously reported (Hou et al., 2020). HDMECs were cultured in endothelial cell growth medium (EGM) composed of endothelial cell basal medium (EBM-2, Lonza, Switzerland) supplemented with EGM-2 MV SingleQuots kit (Lonza, Switzerland), and incubated at 37 °C in a humidified atmosphere containing 5% CO_2_. For the Transwell co-culture system, HMEC-1 or HDMECs were first seeded into the lower chamber of a Transwell insert (Corning, USA) with a 0.4-μm pore size. Depending on the experimental group, Tie2 kinase inhibitor (Tie2i, Abcam, UK), angiogenesis inhibitor (KYP-2047, MCE, USA), Tie2 activator (AKB-9778, MCE, USA), PI3K inhibitor (LY294002, MCE, USA), PI3K activator (740 Y-P, MCE, USA), MEK inhibitor (PD98059, MCE, USA) or STAT3 inhibitor (Stattic, MCE, USA) was added to the medium. Subsequently, either N-DMSCs overexpressing ANGPT1 or psoriatic DMSCs (P-DMSCs) with ANGPT1 knockdown, selected based on the differential expression of Ang1 between N-DMSCs and P-DMSCs, were seeded into the upper chamber. Cells were co-cultured at 37 °C with 5% CO_2_ for 72 h, after which HMEC-1 or HDMECs were collected for subsequent experiments.

### Enzyme-linked immunosorbent assay (ELISA)

The concentration of Ang1 in the medium from the lower chamber of the co-culture system was measured using an Ang1 ELISA Kit (Xitang, China). Absorbance at 450 nm was detected using a microplate reader (Molecular Devices, USA). A standard curve was generated with the standard concentration as the x-axis and the corresponding absorbance as the y-axis, and the Ang1 concentration was calculated based on this curve.

### Cell viability assay

HMEC-1 were seeded into 96-well plates at a density of 5 × 10^3^ cells/well and cultured at 37 °C. Each day, 10 μL of CCK-8 reagent (Boster, China) was added to each well, and cells were incubated for an additional 2 h. Absorbance at 450 nm was measured using a microplate reader (Molecular Devices, USA) for 3 consecutive days to assess cell viability.

### EdU staining assay

The proliferation of HMEC-1 or HDMECs was detected using the EdU Cell Proliferation Assay Kit (RiboBio, China). Cells were seeded into 96-well plates and cultured overnight at 37 °C. Cells were then incubated with 50 μM EdU solution for 2 h, fixed with 4% paraformaldehyde, permeabilized with 0.5% Triton X-100, and stained with Apollo 567. Nuclei were counterstained with Hoechst 33,342 (5 mg/mL). Cells were imaged using a high-content cell imaging analysis system (YOKOGAWA, Japan), and 5 random fields were selected to count EdU-positive cells.

### Transwell cell migration assay

A total of 600 μL of complete MCDB131 growth medium was added to the lower chamber of an 8-μm Transwell insert (Corning, USA). HMEC-1 or HDMECs were resuspended in serum-free MCDB131 medium at a density of 5 × 10^4^ cells/well and seeded into the upper chamber. Cells were allowed to migrate at 37 °C for 24 h. Non-migrated cells inside the upper chamber were removed using a cotton swab. Migrated cells on the lower surface of the membrane were fixed with 4% paraformaldehyde and stained with 0.5% crystal violet (Solarbio, China). Migrated cells were observed using an inverted phase-contrast microscope (Olympus, Japan), and 5 random fields were selected for counting to determine the number of migrated cells.

### Tube formation assay

The *in vitro* tube formation capacity of HMEC-1 or HDMECs was evaluated using Matrigel Basement Membrane Matrix (Corning, USA). A total of 50 μL of pre-melted Matrigel (thawed at 4 °C) was added to each well of a 96-well plate and allowed to solidify at 37 °C for 2 h. Collected HMEC-1 were seeded onto the Matrigel at a density of 2 × 10^4^ cells/well and incubated at 37 °C in a humidified incubator with 5% CO_2_ for 6 h. Vascular-like structures were observed using an inverted phase-contrast microscope (Olympus, Japan). 5 random fields were imaged, and the number of nodes, meshes, junctions, and branches was quantified using ImageJ software.

### Mice

C57BL/6 mice were purchased from the Animal Experiment Center of Shanxi Medical University. All mice were housed under a 12-h light/dark cycle at 21 °C ± 2 °C with free access to standard laboratory food and water. Mice aged 5–8 weeks were used for experiments, and all animal procedures were approved by the Animal Ethics Committee of Shanxi Medical University.

### Induction of the imiquimod (IMQ)-Induced psoriasis-like mouse model

One day before the experiment, the hair on the dorsal skin of mice was shaved using an electric clipper (exposing an area of 2 cm^2^). Mice were then topically treated with 62.5 mg of 5% IMQ cream (Mingxinlidi, China) on the dorsal skin once daily for 5 days to induce psoriasis-like dermatitis. To evaluate the effect of ANGPT1 on psoriasis progression, mice were anesthetized via inhalation of isoflurane (3% for induction, 1%–1.5% for maintenance, in 100% oxygen at a flow rate of 1 L/min) using a calibrated laboratory vaporizer 6 h after each IMQ treatment, prior to intradermal plasmid injection. Subsequently, 25 μL of Entranster™-in vivo reagent (Engreen, China) containing 12.5 μg of pLVX-ANGPT1^high^ or pLKO.1-shANGPT1 plasmids was administered intradermally on the dorsal skin. pLVX-Vehicle and pLKO.1-shNC served as negative controls. A daily PASI-like score was assigned according to the Psoriasis Area and Severity Index (PASI) criteria to assess the severity of skin lesions. On day 6, mice were humanely euthanized by cervical dislocation after being deeply anesthetized with isoflurane (5% inhalation), in accordance with the AVMA Guidelines for the Euthanasia of Animals (2020 Edition). Lesional skin tissues were collected for subsequent analysis.

### Immunohistochemistry

Mouse skin lesions were collected, fixed in 4% paraformaldehyde, and embedded in paraffin. Sections were cut at 4 μm thickness, deparaffinized, rehydrated, and subjected to antigen retrieval in citrate buffer (pH 6.0). After blocking with 5% normal goat serum for 30 min at room temperature, sections were incubated overnight at 4 °C with the following primary antibodies: anti-CD31 (1:1000, Abcam, UK), anti-α-SMA (1:1000, Proteintech, USA), and anti-Ang2 (1:100, HUABIO, China). The next day, sections were incubated with HRP-conjugated secondary antibody for 1 h at room temperature, followed by DAB development, hematoxylin counterstaining, dehydration, and mounting. Images were captured using a light microscope (Olympus, Japan). For each section, 5 random fields were selected, and the percentage of positive staining area relative to the total field area was analyzed using ImageJ software, with consistent thresholds applied across all groups.

### Statistical analysis

GraphPad Prism 8.0 and SPSS 25.0 software were used for statistical analysis. All results are presented as the mean ± standard deviation (SD) and were derived from at least three independent experiments. For normally distributed data, Student’s t-test or Welch’s t-test was used for comparisons between two groups, and one-way or two-way analysis of variance (ANOVA) was used for comparisons among multiple groups; non-parametric tests were used for non-normally distributed data. A *p*-value <0.05 was considered statistically significant. In addition, a post-hoc power analysis for the comparison of ANGPT1 mRNA expression (n = 18 per group) was performed using G*Power 3.1.9.2. Based on a two-tailed independent t-test at α = 0.05, the calculated Cohen’s d was 0.9888, with an achieved statistical power of 0.82 (Supplementary Figure S6), confirming that the sample size was statistically sufficient.

## Results

### Ang1 is upregulated in P-DMSCs, induced by IL-17A/TNF-α, and regulates HMEC-1 via paracrine signaling

To identify key angiogenesis-related genes dysregulated in P-DMSCs, we first examined the expression of 15 candidate genes in 6 N-DMSCs and 6 P-DMSCs. qRT-PCR analysis revealed that ANGPT1 and VCAM1 were significantly upregulated in P-DMSCs, while FGF2, DLL4, JAG1, PDGFA, PDGFB, and other genes showed no significant changes (Supplementary Figure S1). Given its role as a secreted ligand, ANGPT1 was selected as a candidate effector molecule for further investigation.

**FIGURE 1:**
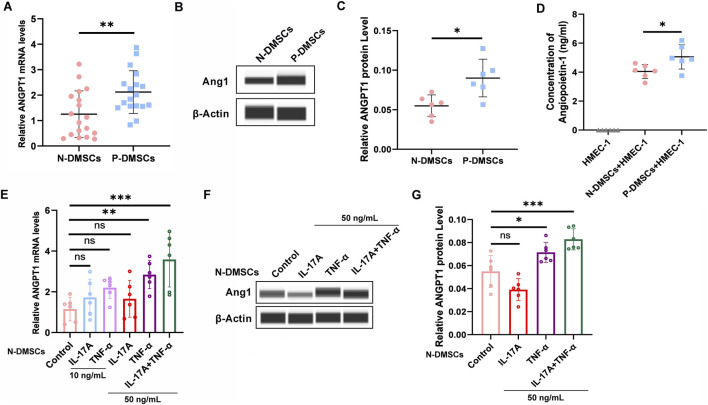
Expression Levels of Ang1 in N-DMSCs and P-DMSCs. **(A)** ANGPT1 mRNA levels in N-DMSCs and P-DMSCs analyzed by qRT-PCR (n = 18 per group). **(B,C)** Ang1 protein levels in N-DMSCs and P-DMSCs detected by Western blotting (expression levels normalized to β-actin; n = 6 per group). **(D)** Ang1 levels in the supernatant of HMEC-1 co-cultured with N-DMSCs or P-DMSCs measured by ELISA (n = 6 per group). **(E)** ANGPT1 mRNA levels in N-DMSCs stimulated with IL-17A (10, 50 ng/mL), TNF-α (10, 50 ng/mL), or a combination of both for 48 h analyzed by qRT-PCR (n = 6 per group). **(F,G)** Ang1 protein levels in N-DMSCs under the same stimulation conditions detected by Western blotting (expression levels normalized to β-actin; n = 6 per group). All experiments were performed in triplicate. ns *p* > 0.05, *p < 0.05, **p < 0.01, ***p < 0.001.

Based on this screening, we next validated Ang1 expression between N-DMSCs and P-DMSCs in an expanded cohort. qRT-PCR analysis of 18 N-DMSC and 18 P-DMSC samples showed that ANGPT1 mRNA levels were significantly higher in P-DMSCs than in N-DMSCs (*p* < 0.01, Figure 1A). Western blotting further confirmed that Ang1 protein expression was also significantly increased in P-DMSCs compared with N-DMSCs (*p* < 0.05, Figures 1B,C), indicating that elevated Ang1 expression in P-DMSCs is consistent at both the transcriptional and translational levels.

Considering that DMSCs may regulate target cell function via paracrine signaling, we further explored the effect of DMSC-secreted Ang1 on HMEC-1. N-DMSCs or P-DMSCs were co-cultured with HMEC-1, and ELISA was used to detect Ang1 levels in the HMEC-1 culture supernatant. Results showed that Ang1 concentrations were significantly higher in the supernatant of HMEC-1 co-cultured with P-DMSCs than in those co-cultured with N-DMSCs (*p* < 0.05, Figure 1D). Importantly, no Ang1 was detected in the supernatant of HMEC-1 cultured alone, ruling out the possibility of endogenous Ang1 synthesis by HMEC-1 and confirming that all Ang1 in the co-culture supernatant was derived from DMSCs (Figure 1D).

To explore the upstream signals responsible for Ang1 upregulation in P-DMSCs, we stimulated N-DMSCs with the psoriasis-associated inflammatory cytokines IL-17A and TNF-α. qRT-PCR analysis showed that IL-17A alone had no significant effect on ANGPT1 expression, while TNF-α alone significantly upregulated ANGPT1 mRNA levels. Notably, combined stimulation with IL-17A and TNF-α resulted in a further increase compared with TNF-α alone, indicating a synergistic effect (Figure 1E). Western blotting confirmed corresponding changes in Ang1 protein expression (Figures 1F,G). These findings suggest that inflammatory cytokines present in the psoriatic microenvironment, particularly TNF-α, serve as upstream signals that directly induce ANGPT1 expression in DMSCs.

### Overexpression of ANGPT1 in N-DMSCs promotes HMEC-1 angiogenesis

**FIGURE 2:**
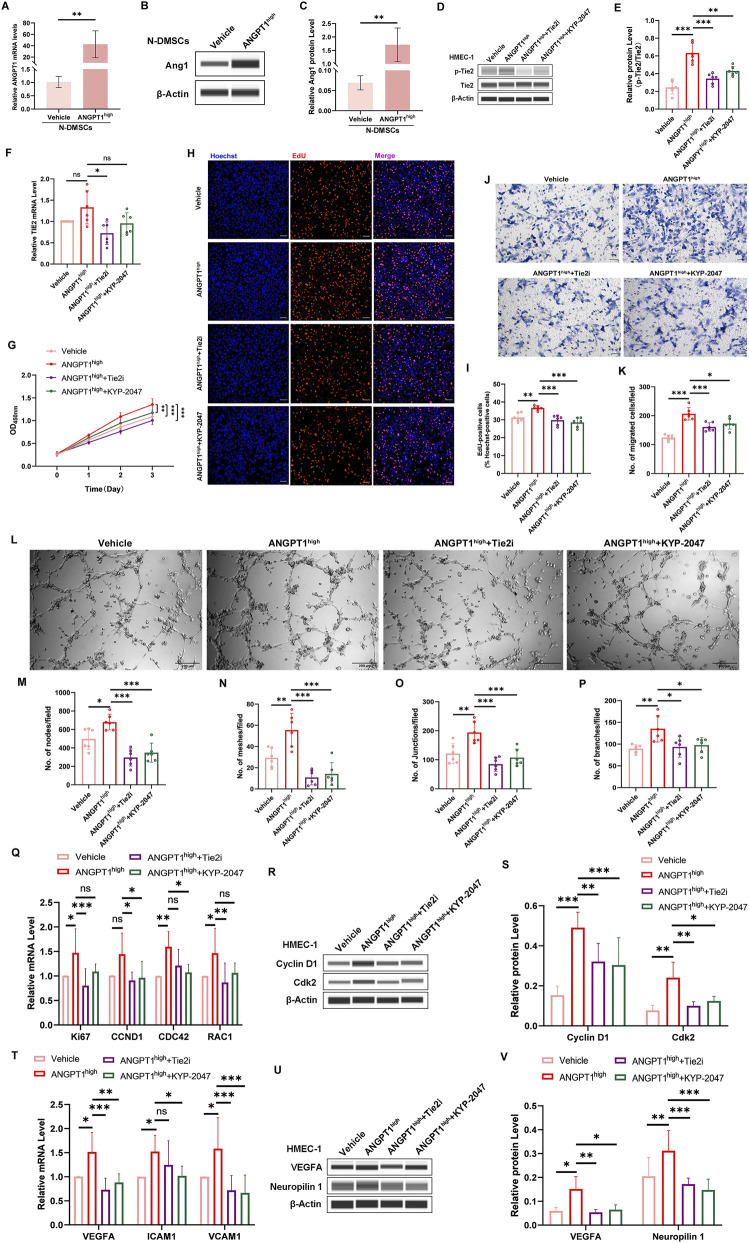
ANGPT1 Overexpression in N-DMSCs Promotes Tie2 Activation and HMEC-1 Angiogenesis. **(A)** ANGPT1 mRNA levels in N-DMSCs transfected with pLVX-ANGPT1^high^ analyzed by qRT-PCR. **(B,C)** Ang1 protein levels in N-DMSCs detected by Western blotting. **(D,E)** Tie2 and p-Tie2 protein levels in HMEC-1 co-cultured with N-DMSCs transfected with pLVX-ANGPT1^high^ detected by Western blotting. **(F)** TIE2 mRNA levels in HMEC-1 analyzed by qRT-PCR. **(G)** HMEC-1 viability measured by CCK-8 assay. **(H,I)** Percentage of EdU-positive HMEC-1 (scale bar = 100 μm). **(J,K)** Numbers of migrated HMEC-1 detected by Transwell assay (scale bar = 50 μm). **(L)** Phase-contrast micrographs of vascular-like structures formed by HMEC-1 (scale bar = 200 μm), and quantitative analysis of nodes **(M)**, meshes **(N)**, junctions **(O)**, and branches **(P)** of vascular-like structures. **(Q,T)** mRNA levels of proliferation-related genes (Ki67, CCND1, CDC42), migration-related genes (RAC1), and angiogenesis-related genes (VEGFA, ICAM1, VCAM1) analyzed by qRT-PCR. **(R,S,U,V)** Protein levels of proliferation-related genes (Cyclin D1, Cdk2) and angiogenesis-related genes (VEGFA, Neuropilin 1) detected by Western blotting. All protein levels normalized to β-actin. All experiments were performed in triplicate (n = 6 per group). ns *p* > 0.05, *p < 0.05, **p < 0.01, ***p < 0.001.

Given that high Ang1 expression in P-DMSCs may contribute to the pathological progression of psoriasis by modulating the angiogenic microenvironment in lesional tissues, we overexpressed ANGPT1 in N-DMSCs to investigate its regulatory effect on HMEC-1 function. N-DMSCs were transfected with pLVX-Vehicle or pLVX-ANGPT1^high^ plasmids. qRT-PCR and Western blotting confirmed that pLVX-ANGPT1^high^ significantly increased Ang1 mRNA and protein expression in N-DMSCs (Figures 2A–C), verifying the successful establishment of the ANGPT1-overexpression model.

To determine whether DMSC-derived Ang1 exerts its effects via the Tie2 receptor on HMEC-1, transfected N-DMSCs were co-cultured with HMEC-1, and additional groups treated with Tie2 kinase inhibitor (Tie2i) or angiogenesis inhibitor (KYP-2047) were included to detect Tie2 expression and phosphorylation in HMEC-1. Compared with the Vehicle group, the ANGPT1^high^ group showed significantly increased p-Tie2 levels, while Tie2i and KYP-2047 treatment significantly reduced p-Tie2 levels (Figures 2D,E). For TIE2 mRNA levels, only the Tie2i group showed a significant decrease compared with the ANGPT1^high^ group, with no significant differences observed in other groups (Figure 2F). These results suggest that Ang1 primarily exerts its effects by activating Tie2 phosphorylation and that inhibitors of Tie2 kinase activity and angiogenesis-related pathways can block this activation.

Next, we investigated the effects of ANGPT1-overexpressing N-DMSCs on HMEC-1 viability, proliferation, migration, and tube formation. CCK-8 assays showed that HMEC-1 viability was significantly higher in the ANGPT1^high^ group than in the Vehicle group, while Tie2i and KYP-2047 treatment reduced viability compared with the ANGPT1^high^ group (Figure 2G). EdU staining revealed a significant increase in the number of EdU-positive cells in the ANGPT1^high^ group compared with the Vehicle group, and this increase was reversed by Tie2i or KYP-2047 treatment (Figures 2H,I). Similarly, Transwell migration assays showed that the number of migrated HMEC-1 was significantly higher in the ANGPT1^high^ group than in the Vehicle group, with Tie2i and KYP-2047 treatment reducing migration compared with the ANGPT1^high^ group (Figures 2J,K). Tube formation assays demonstrated that the number of nodes, meshes, junctions, and branches of vascular-like structures was higher in the ANGPT1^high^ group than in the Vehicle group, and these parameters were significantly reduced in the Tie2i and KYP-2047 groups compared with the ANGPT1^high^ group (Figures 2L–P). To further explore the molecular mechanisms, we detected the expression of function-related genes in HMEC-1. Compared with the Vehicle group, the ANGPT1^high^ group showed significantly increased mRNA and protein expression of proliferation-related genes (Ki67, Cyclin D1, CDC42, CDK2), migration-related genes (RAC1), and angiogenesis-related genes (VEGFA, ICAM1, VCAM1, NRP1). Tie2i or KYP-2047 treatment significantly reduced the expression of these genes compared with the ANGPT1^high^ group (Figures 2Q–V). Collectively, these results indicate that DMSC-secreted Ang1 can significantly enhance HMEC-1 viability, promote proliferation and migration, and increase tube formation capacity, and this regulatory effect depends on the activation of the Tie2 receptor and angiogenesis-related pathways.

In addition, we co-cultured successfully transfected N-DMSCs with primary HDMECs to further validate the pro-angiogenic effect of ANGPT1-overexpressing N-DMSCs. The results were consistent with those obtained from HMEC-1 experiments. Co-culture with ANGPT1-overexpressing N-DMSCs significantly enhanced the proliferation (Supplementary Figures S2A,B), migration (Supplementary Figures S2C,D), and tube formation (Supplementary Figures S2E–I) of HDMECs. Moreover, these pro-angiogenic effects were completely reversed by treatment with Tie2i (Supplementary Figure S2). These findings further confirm that ANGPT1-overexpressing N-DMSCs exert a significant pro-angiogenic effect.

### Knockdown of ANGPT1 in P-DMSCs inhibits HMEC-1 angiogenesis

**FIGURE 3:**
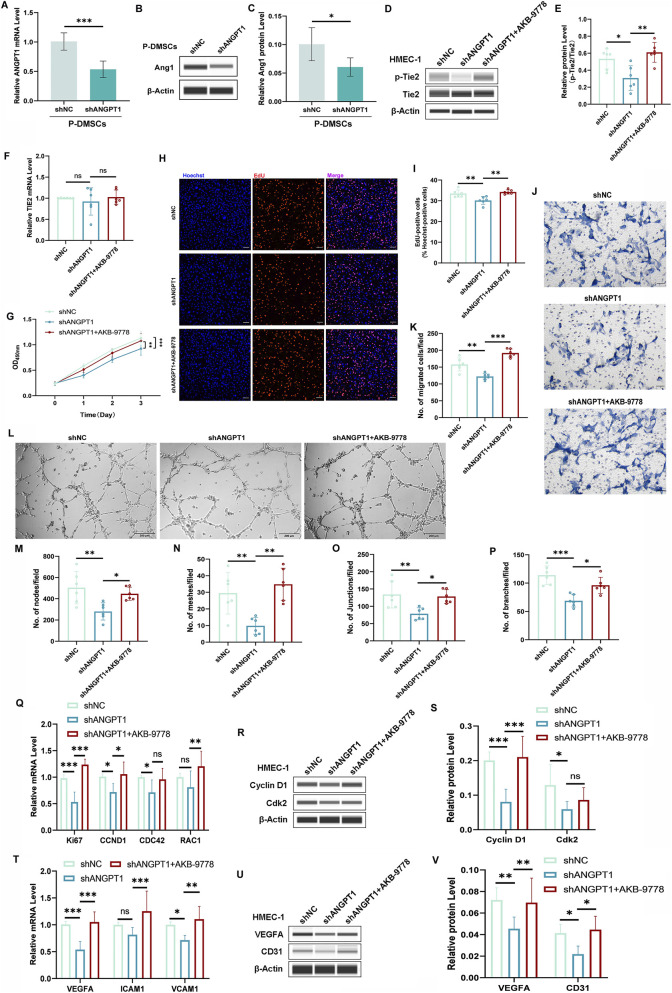
ANGPT1 Knockdown in P-DMSCs Inhibits Tie2 Activation and HMEC-1 Angiogenesis. **(A)** ANGPT1 mRNA levels in P-DMSCs transfected with pLKO.1-shANGPT1 analyzed by qRT-PCR. **(B,C)** Ang1 protein levels in P-DMSCs detected by Western blotting. **(D,E)** Tie2 and p-Tie2 protein levels in HMEC-1 co-cultured with P-DMSCs transfected with pLKO.1-shANGPT1 detected by Western blotting. **(F)** TIE2 mRNA levels in HMEC-1 analyzed by qRT-PCR. **(G)** HMEC-1 viability measured by CCK-8 assay. **(H,I)** Percentage of EdU-positive HMEC-1 cells (scale bar = 100 μm). **(J,K)** Numbers of migrated HMEC-1 detected by Transwell assay (scale bar = 50 μm). **(L)** Phase-contrast micrographs of vascular-like structures formed by HMEC-1 (scale bar = 200 μm), and quantitative analysis of nodes **(M)**, meshes **(N)**, junctions **(O)**, and branches **(P)** of vascular-like structures. **(Q,T)** mRNA levels of proliferation-related genes (Ki67, CCND1, CDC42), migration-related genes (RAC1), and angiogenesis-related genes (VEGFA, ICAM1, VCAM1) analyzed by qRT-PCR. **(R,S,U,V)** Protein levels of proliferation-related genes (Cyclin D1, Cdk2) and angiogenesis-related genes (VEGFA, CD31) detected by Western blotting. All protein levels normalized to β-actin. All experiments were performed in triplicate (n = 6 per group). ns *p* > 0.05, *p < 0.05, **p < 0.01, ***p < 0.001.

To further confirm Ang1/Tie2 axis-mediated signaling between DMSCs and HMEC-1, we performed reverse validation by knocking down ANGPT1 in P-DMSCs. P-DMSCs were transfected with pLKO.1-shANGPT1 (to establish an ANGPT1-knockdown model) or pLKO.1-shNC (negative control). qRT-PCR and Western blotting showed that shANGPT1 significantly reduced Ang1 mRNA and protein expression in P-DMSCs (Figures 3A–C), confirming the successful establishment of the ANGPT1-knockdown model.

Transfected P-DMSCs were then co-cultured with HMEC-1 to detect Tie2 expression and phosphorylation in HMEC-1, with an additional group treated with Tie2 activator (AKB-9778). No significant differences in TIE2 mRNA levels were observed among groups (Figure 3F); however, p-Tie2 expression was significantly lower in the shANGPT1 group than in the shNC group (Figures 3D,E). AKB-9778 treatment significantly restored p-Tie2 levels in the shANGPT1 group (Figures 3D,E), confirming that P-DMSC-secreted Ang1 can specifically activate Tie2 phosphorylation in HMEC-1 and that this activation can be reversed by a Tie2 activator.

We further investigated the effects of ANGPT1 knockdown in P-DMSCs on HMEC-1 viability, proliferation, migration, and tube formation. CCK-8 assays showed that HMEC-1 viability was significantly lower in the shANGPT1 group than in the shNC group, and AKB-9778 treatment significantly increased viability compared with the shANGPT1 group (Figure 3G), indicating that Tie2 activation can reverse the reduction in cell viability caused by ANGPT1 knockdown. EdU staining revealed a significant decrease in the number of EdU-positive cells in the shANGPT1 group compared with the shNC group, and AKB-9778 treatment reversed this decrease (Figures 3H,I). Transwell migration assays showed that the number of migrated HMEC-1 was significantly lower in the shANGPT1 group than in the shNC group, and AKB-9778 treatment significantly reversed this inhibitory effect (Figures 3J,K). Tube formation assays demonstrated that shANGPT1 significantly reduced the number of nodes, meshes, junctions, and branches of vascular-like structures in HMEC-1 compared with the shNC and AKB-9778 groups (Figures 3L–P). To clarify the underlying molecular mechanisms, we detected the mRNA and protein expression of proliferation-related genes (Ki67, Cyclin D1, CDC42, CDK2), migration-related genes (RAC1), and angiogenesis-related genes (VEGFA, ICAM1, VCAM1, CD31) in the three groups. Results showed that the expression of these genes was significantly lower in the shANGPT1 group than in the shNC group, and AKB-9778 treatment significantly increased their expression compared with the shANGPT1 group (Figures 3Q–V), which was consistent with the results of HMEC-1 functional assays. Collectively, these findings indicate that reducing Ang1 secretion by DMSCs significantly decreases HMEC-1 viability, inhibits proliferation and migration, and impairs vascular-like structure formation, highlighting the core regulatory role of the Ang1/Tie2 axis in DMSC-HMEC-1 signaling and vascular abnormalities in psoriasis.

Similarly, the inhibitory effect induced by ANGPT1 knockdown was also validated in primary HDMECs. Co-culture with ANGPT1-knockdown P-DMSCs significantly suppressed the proliferation (Supplementary Figures S3A,B), migration (Supplementary Figures S3C,D), and tube formation (Supplementary Figures S3E–I) of HDMECs, and these inhibitory effects were partially rescued by treatment with AKB-9778 (Supplementary Figure S3). Although some inter-individual variability was observed among HDMEC samples, the overall trends were highly consistent with our findings in HMEC-1, further confirming that reducing Ang1 secretion significantly attenuates the pro-angiogenic effect of N-DMSCs.

### Upregulation of Ang1 in N-DMSCs promotes HMEC-1 angiogenesis via activation of the PI3K/AKT pathway

**FIGURE 4:**
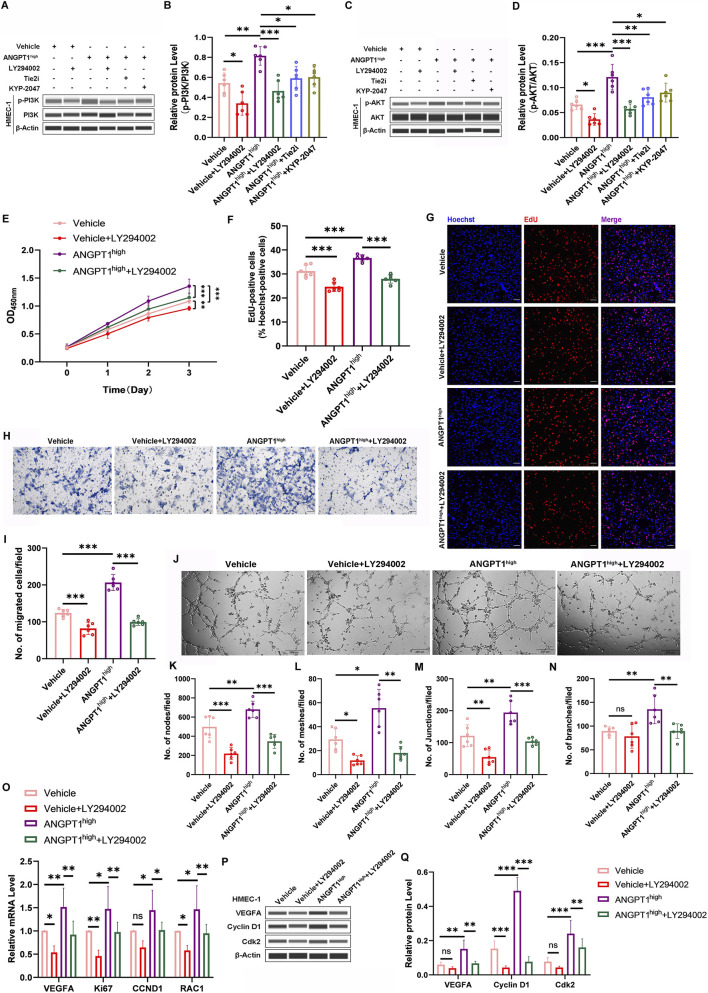
ANGPT1-Overexpressing N-DMSCs Promote HMEC-1 Angiogenesis via the PI3K/AKT Pathway. **(A–D)** Protein levels of p-PI3K **(A,B)** and p-AKT **(C,D)** in HMEC-1 co-cultured with N-DMSCs transfected with pLVX-ANGPT1^high^ (with or without LY294002 treatment) detected by Western blotting. **(E)** HMEC-1 viability measured by CCK-8 assay. **(F,G)** Percentage of EdU-positive HMEC-1 cells (scale bar = 100 μm). **(H,I)** Numbers of migrated HMEC-1 detected by Transwell assay (scale bar = 50 μm). **(J)** Phase-contrast micrographs of vascular-like structures formed by HMEC-1 (scale bar = 200 μm), and quantitative analysis of nodes **(K)**, meshes **(L)**, junctions **(M)**, and branches **(N)** of vascular-like structures. **(O)** mRNA levels of proliferation-related genes (Ki67, CCND1), migration-related genes (RAC1), and angiogenesis-related genes (VEGFA) analyzed by qRT-PCR. **(P,Q)** Protein levels of proliferation-related genes (Cyclin D1, Cdk2) and angiogenesis-related genes (VEGFA) detected by Western blotting. All protein levels normalized to β-actin. All experiments were performed in triplicate (n = 6 per group). ns *p* > 0.05, *p < 0.05, **p < 0.01, ***p < 0.001.

Our previous studies suggested that the Ang1/Tie2 axis exerts its proangiogenic effects primarily via the PI3K/AKT signaling pathway (Li et al., 2025). To verify whether DMSC-secreted Ang1 regulates HMEC-1 function via this pathway, we first detected PI3K and AKT activation in HMEC-1 co-cultured with ANGPT1-overexpressing N-DMSCs. Western blotting showed that compared with the Vehicle group, the ANGPT1^high^ group exhibited significantly increased p-PI3K and p-AKT levels in HMEC-1. In contrast, Tie2i and KYP-2047 treatment significantly reduced p-PI3K and p-AKT levels compared with the ANGPT1^high^ group (Figures 4A–D), indicating that DMSC-secreted Ang1 can effectively activate the PI3K/AKT pathway in HMEC-1 and that this activation depends on the Tie2 receptor.

To further confirm the core role of the PI3K/AKT pathway in regulating HMEC-1 function, a PI3K inhibitor (LY294002) was added to the co-culture system. Results showed that LY294002 significantly reduced p-PI3K and p-AKT levels in both the Vehicle and ANGPT1^high^ groups compared with the same groups without LY294002 treatment (Figures 4A–D), confirming the effective inhibition of PI3K/AKT pathway activation by LY294002. We then investigated the effects of LY294002 on HMEC-1 viability, proliferation, migration, and tube formation. CCK-8 assays showed that LY294002 treatment significantly reduced HMEC-1 viability in both the Vehicle and ANGPT1^high^ groups compared with the same groups without LY294002 (Figure 4E). EdU staining revealed a significant decrease in the number of EdU-positive cells in the Vehicle and ANGPT1^high^ groups following LY294002 treatment (Figures 4F,G). Transwell migration assays showed that LY294002 treatment significantly reduced the number of migrated HMEC-1 compared with the same groups without LY294002 (Figures 4H,I). Tube formation assays demonstrated that LY294002 treatment significantly reduced the number of nodes, meshes, junctions, and branches of vascular-like structures compared with the same groups without LY294002, except for the number of branches, which showed no significant difference between the Vehicle + LY294002 group and the Vehicle group alone (Figures 4J–N). Correspondingly, we detected the expression of proliferation-, migration-, and angiogenesis-related genes in HMEC-1. In the Vehicle group, LY294002 treatment significantly reduced the mRNA levels of Ki67, RAC1, and VEGFA but had no effect on CCND1 mRNA levels; it also significantly reduced Cyclin D1 protein levels but had no effect on Cdk2 and VEGFA protein levels. In contrast, in the ANGPT1^high^ group, LY294002 treatment significantly reduced the mRNA and protein levels of all tested genes (Figures 4O–Q). These results indicate that DMSC-secreted Ang1 can activate the Tie2 receptor, thereby triggering the PI3K/AKT pathway, which ultimately positively regulates HMEC-1 viability, proliferation, migration, and tube formation.

In addition to the PI3K/AKT pathway, we further examined the activation of the MAPK/ERK and STAT3 pathways downstream of the Ang1/Tie2 axis. After co-culturing ANGPT1-overexpressing N-DMSCs with HMEC-1, the levels of p-ERK1/2 and p-STAT3 in HMEC-1 were significantly increased; treatment with Tie2i significantly blocked these increases, indicating that the activation of ERK and STAT3 depends on the Tie2 receptor (Supplementary Figures S4A–D). Functional inhibition experiments showed that the MEK inhibitor PD98059 and the STAT3 inhibitor Stattic both partially suppressed ANGPT1 overexpression-induced HMEC-1 proliferation (Supplementary Figures S4E,F), migration (Supplementary Figures S4G,H), and tube formation (Supplementary Figures S4I–M), albeit to a lesser extent than the PI3K inhibitor LY294002. These results suggest that the Ang1/Tie2 axis promotes angiogenesis by activating multiple downstream signaling pathways, with the PI3K/AKT pathway playing a primary role, while the MAPK/ERK and STAT3 pathways may participate as ancillary signaling axes.

### Downregulation of Ang1 in P-DMSCs reduces HMEC-1 angiogenesis via inhibition of the PI3K/AKT pathway

**FIGURE 5:**
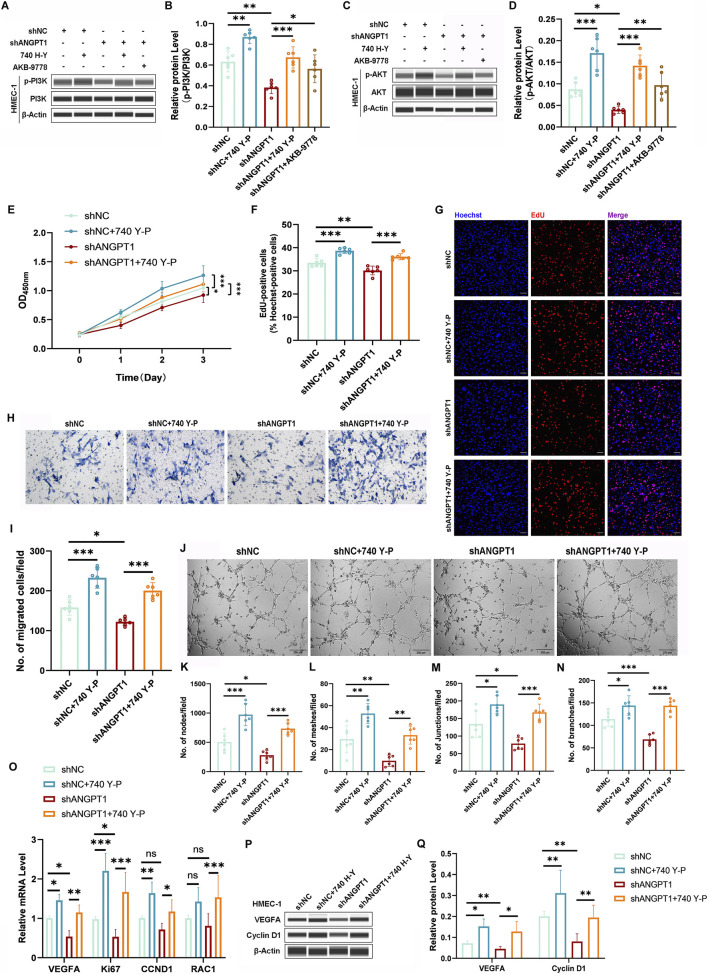
ANGPT1-Knockdown P-DMSCs Inhibit HMEC-1 Angiogenesis via the PI3K/AKT Pathway. **(A–D)** Protein levels of p-PI3K **(A,B)** and p-AKT **(C,D)** in HMEC-1 co-cultured with P-DMSCs transfected with pLKO.1-shANGPT1 (with or without 740 Y-P treatment) detected by Western blotting. **(E)** HMEC-1 viability measured by CCK-8 assay. **(F,G)** Percentage of EdU-positive HMEC-1 cells (scale bar = 100 μm). **(H,I)** Numbers of migrated HMEC-1 detected by Transwell assay (scale bar = 50 μm). **(J)** Phase-contrast micrographs of vascular-like structures formed by HMEC-1 (scale bar = 200 μm), and quantitative analysis of nodes **(K)**, meshes **(L)**, junctions **(M)**, and branches **(N)** of vascular-like structures. **(O)** mRNA levels of proliferation-related genes (Ki67, CCND1), migration-related genes (RAC1), and angiogenesis-related genes (VEGFA) analyzed by qRT-PCR. **(P,Q)** Protein levels of Cyclin D1 and VEGFA detected by Western blotting. All protein levels normalized to β-actin. All experiments were performed in triplicate (n = 6 per group). ns *p* > 0.05, *p < 0.05, **p < 0.01, ***p < 0.001.

To enhance the rigor of our experiments, we simultaneously investigated the co-culture of ANGPT1-knockdown P-DMSCs with HMEC-1. As shown in Figures 5A–D, shANGPT1 significantly reduced p-PI3K and p-AKT levels in HMEC-1, and AKB-9778 treatment significantly reversed the inhibitory effect of shANGPT1 on p-PI3K and p-AKT expression (Figures 5A–D), confirming that P-DMSC-secreted Ang1 can promote the activation of the downstream PI3K/AKT pathway in HMEC-1.

To further confirm the regulatory effect of the PI3K/AKT pathway on HMEC-1 function, a rescue experiment was performed using a PI3K activator (740 Y-P). Results showed that 740 Y-P effectively restored p-PI3K and p-AKT expression, which was inhibited by shANGPT1 (Figures 5A–D). CCK-8 assays showed that 740 Y-P treatment increased HMEC-1 viability in both the shNC and shANGPT1 groups compared with the same groups without 740 Y-P (Figure 5E). EdU staining and Transwell migration assays showed that 740 Y-P treatment increased the number of EdU-positive cells and migrated HMEC-1 in the shNC +740 Y-P and shANGPT1 + 740 Y-P groups compared with the shNC and shANGPT1 groups alone (Figures 5F–I). Tube formation assays demonstrated that 740 Y-P treatment significantly increased the number of nodes, meshes, junctions, and branches of HMEC-1-derived vascular-like structures (Figures 5J–N). Consistent with the functional assay results, qRT-PCR and Western blotting showed that 740 Y-P treatment significantly increased the mRNA and protein expression of VEGFA and Cyclin D1, as well as the mRNA levels of Ki67 and RAC1. The only exception was RAC1 mRNA expression, which showed no significant difference in the shNC group before and after 740 Y-P treatment (Figures 5O–Q). These results indicate that reducing Ang1 secretion by DMSCs inhibits PI3K/AKT pathway activity, and activation of this pathway can reverse the shANGPT1-induced reduction in HMEC-1 viability, proliferation, migration, and tube formation capacity.

### Ang1 enhances psoriasis-like lesions in the IMQ-induced psoriasis mouse model

**FIGURE 6:**
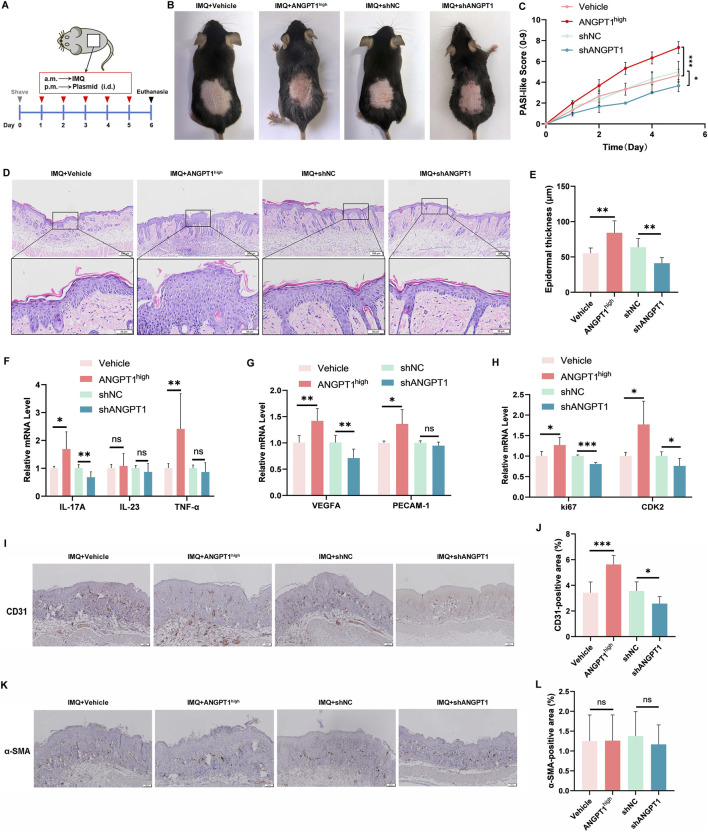
Ang1 Enhances Psoriasis-Like Lesions and Impairs Vascular Maturity in the IMQ-Induced Psoriasis Mouse Model. **(A)** Experimental protocol. **(B)** Clinical images of dorsal skin lesions in IMQ-treated mice treated with pLVX-Vehicle, pLVX-ANGPT1^high^, pLKO.1-shNC, or pLKO.1-shANGPT1. **(C)** PASI-like scores. **(D)** Hematoxylin-eosin (HE) staining of lesional skin (scale bar = 200 μm for ×4 magnification and scale bar = 50 μm for ×20 magnification). **(E)** Epidermal thickness quantification. **(F–H)** mRNA levels of IL-17A, IL23, and TNF-α **(F)**, VEGFA and PECAM-1 **(G)**, and Ki67 and CDK2 **(H)** analyzed by qRT-PCR. **(I)** Immunohistochemical staining of CD31 in lesional skin (scale bar = 100 μm). **(J)** Quantification of CD31-positive area. **(K)** Immunohistochemical staining of α-SMA in lesional skin (scale bar = 100 μm). **(L)** Quantification of α-SMA-positive area. All experiments were performed in triplicate (n = 3 per group). ns *p* > 0.05, *p < 0.05, **p < 0.01, ***p < 0.001.

To further evaluate the regulatory effect of Ang1 on angiogenesis in psoriasis, we established a mouse model of dorsal psoriasis-like lesions and verified the role of Ang1 in disease progression via *in vivo* experiments. During the experiment, mice were topically treated with IMQ on the dorsal skin every morning to induce lesions, and transfection reagents containing the corresponding plasmids (pLVX-Vehicle, pLVX-ANGPT1^high^, pLKO.1-shNC, or pLKO.1-shANGPT1) were locally applied to the same site in the afternoon (with an interval of 6 h). The experimental protocol is shown in Figure 6A. To evaluate *in vivo* transfection efficiency, we measured Ang1 expression in lesional skin tissues. qRT-PCR analysis showed that ANGPT1 mRNA levels were significantly increased in the ANGPT1^high^ group and decreased in the shANGPT1 group compared with their respective controls (Supplementary Figure S5A). Western blot analysis confirmed corresponding changes in Ang1 protein levels (Supplementary Figures S5B,C). After IMQ treatment, typical psoriasis-like lesions (erythema and scaling) were observed on the dorsal skin of mice in all groups; however, significant differences in lesion severity were observed among the treatment groups (Figure 6B). Compared with the Vehicle group, local application of the ANGPT1^high^ plasmid exacerbated the psoriasis-like phenotype in IMQ-treated mice, while the shANGPT1 group showed improved skin condition compared with the shNC group (Figure 6B). Consistent with visual observations, the ANGPT1^high^ group showed a significantly higher PASI-like score than the Vehicle group on day 5, while the shANGPT1 group showed a lower score than the shNC group (Figure 6C), confirming a positive correlation between ANGPT1 expression and psoriasis lesion severity. Further histopathological analysis revealed significant differences in the epidermal thickness of lesional skin among groups (Figures 6D,E). The epidermal thickness of IMQ-treated mice in the ANGPT1^high^ group was significantly greater than that in the Vehicle group, suggesting that ANGPT1 overexpression promotes abnormal epidermal cell proliferation. In contrast, the epidermal thickness of the shANGPT1 group was significantly thinner than that of the shNC group, indicating that inhibiting ANGPT1 expression alleviates excessive epidermal proliferation. These findings were fully consistent with the changes in lesion phenotype severity.

Subsequently, total mRNA was extracted from mouse lesional tissues to detect the expression of inflammatory factors. Compared with the Vehicle group, the ANGPT1^high^ group showed significantly increased expression of the inflammatory factors IL17A and TNF-α, with no significant difference in IL23 mRNA expression. Compared with the shNC group, the shANGPT1 group showed significantly decreased IL17A mRNA expression in lesional tissues, with no significant differences in TNF-α and IL23 expression (Figure 6F). These results suggest that ANGPT1 overexpression exacerbates local inflammatory responses primarily by upregulating IL-17A and TNF-α, while inhibiting ANGPT1 specifically reduces IL-17A-mediated inflammation. We then detected the expression of proliferation- and angiogenesis-related genes. Compared with the Vehicle group, the ANGPT1^high^ group showed significantly increased mRNA expression of Ki67, CDK2, VEGFA, and PECAM-1. Compared with the shNC group, the shANGPT1 group showed significantly decreased expression of all tested genes except PECAM-1, which showed no significant difference (Figures 6G,H). These results indicate that ANGPT1 can positively regulate the transcription of proliferation- and angiogenesis-related genes in lesional tissues. In summary, the *in vivo* experimental results were consistent with the severity of psoriasis lesions and the conclusions from *in vitro* cell experiments, suggesting that ANGPT1 upregulation promotes the exacerbation of inflammatory responses, abnormal cell proliferation, and increased angiogenesis in psoriasis lesions, while inhibiting ANGPT1 expression alleviates these pathological changes. These findings further confirm the key role of Ang1 in the pathological progression of psoriasis and provide *in vivo* experimental evidence for the subsequent targeting of Ang1 to intervene in vascular abnormalities in psoriasis.

To evaluate vascular characteristics and maturity in lesional skin of different treatment groups, we performed immunohistochemical staining for CD31 (endothelial cells), α-SMA (pericytes), and Ang2. As shown in Figures 6I,J, compared with the Vehicle group, the ANGPT1^high^ group exhibited a significant increase in CD31^+^ vessel density, while the shANGPT1 group showed a significant decrease. α-SMA staining revealed no significant differences in α-SMA^+^ positive area among the groups (Figures 6K,L). Immunohistochemical analysis of Ang2 showed that the Ang2-positive area was significantly increased in the ANGPT1^high^ group compared with the Vehicle group, and significantly decreased in the shANGPT1 group compared with the shNC group (Supplementary Figures S5D,E). Taken together, these results indicate that ANGPT1 overexpression increases vessel density but does not proportionally increase pericyte coverage, accompanied by elevated Ang2 expression, suggesting impaired maturation of newly formed vessels. Conversely, ANGPT1 knockdown reduces vessel density, retains relatively more pericyte coverage, and decreases Ang2 expression, indicating a trend toward vascular stabilization.

## Discussion

The pathological vicious cycle formed by chronic inflammation and abnormal vascular hyperplasia is the core mechanism underlying the persistence of psoriatic lesions (Li et al., 2021). Specifically, abnormal vascular hyperplasia is characterized by increased numbers and lengths of capillaries, accompanied by the infiltration of mixed immune cells around blood vessels (Bolognia et al., 2018). These overproliferated microvessels not only supply nutrients to lesional tissues but also serve as channels for inflammatory cell infiltration. Furthermore, inflammatory factors further drive angiogenesis, ultimately exacerbating the abnormal proliferation of epidermal keratinocytes (Chen et al., 2023). As key regulatory cells in the skin microenvironment, DMSCs can modulate the function of surrounding cells via paracrine cytokines (Correa-Araujo et al., 2023); however, their specific mechanism of action in psoriasis-associated vascular abnormalities remains incompletely understood. Focusing on Ang1, a critical regulator of angiogenesis, this study systematically delineated the complete mechanism through a combination of *in vitro* cell experiments and *in vivo* animal model validation: highly expressed Ang1 in P-DMSCs activates the Tie2 receptor on the surface of HMEC-1, triggers the PI3K/AKT signaling pathway, and thereby promotes HMEC-1 proliferation, migration, and tube formation, ultimately contributing to the pathological progression of psoriasis. These findings provide pivotal evidence for understanding the molecular basis of vascular abnormalities in psoriasis and developing targeted intervention strategies.

The papillary dermis is an active site for intercellular communication during psoriasis-related inflammation (Gniadecki et al., 2023; Braverman, 1983). Studies have shown that significant abnormal angiogenesis in psoriatic lesions is closely associated with intercellular signaling in the dermal microenvironment (Zhang and Wu, 2024). Intercellular communication can occur directly via gap junctions or indirectly via soluble factors and extracellular vesicles (Kalluri and LeBleu, 2020). Single-cell RNA sequencing and spatial transcriptome sequencing analyses of lesional and non-lesional tissues from psoriatic patients and skin tissues from healthy individuals have indicated that stromal cells in the papillary dermis make important contributions to the psoriatic lesion microenvironment (Zhang et al., 2025). DMSCs are the main component of stromal cells, and our previous studies have found that P-DMSCs exhibit dysregulated expression of angiogenesis-related genes and possess the ability to promote EC angiogenesis (Niu et al., 2016a; Niu et al., 2016b; Zhou et al., 2021). Traditionally, research on angiogenesis in psoriasis has focused primarily on the VEGF/VEGFR pathway (Zhang J. et al., 2023); however, anti-VEGF therapy has not been effective in psoriasis, suggesting the existence of other key and synergistic regulatory mechanisms. The Ang1/Tie2 axis, an essential system for vascular homeostasis and remodeling, has recently attracted attention in psoriasis research. This study focused on Ang1 as a core effector molecule and identified its specific overexpression in P-DMSCs. Ang1 not only serves as a molecular marker of DMSCs functional abnormalities in psoriasis but also acts as a key bridge linking dermal microenvironment disorders and abnormal angiogenesis in psoriasis. More importantly, HMEC-1 does not synthesize Ang1 endogenously, and all Ang1 in the supernatant is derived from DMSCs. Furthermore, Ang1 levels in the supernatant of the P-DMSCs group were significantly higher than those in the N-DMSCs group. These results directly rule out the interference of HMEC-1-derived Ang1, confirming that P-DMSCs can secrete Ang1 via paracrine signaling, providing continuous angiogenic signals to ECs in lesional tissues and thus serving as a potential source of signals driving vascular abnormalities in psoriasis.

Our results show that Ang1 can activate Tie2 phosphorylation without upregulating receptor expression, which is consistent with the classic mechanism in normal vascular development, where Ang1/Tie2 axis signaling depends on receptor phosphorylation (Wang et al., 2025). The pathophysiological progression of psoriasis is regulated by abnormal dermal papillary vascular structure, and vascular changes precede epidermal hyperplasia in psoriatic lesions (Lee et al., 2021). Angiogenesis involves a series of processes, including cell proliferation, migration, and tube formation, which provide sufficient oxygen, nutrients, and growth factors to target tissues (Hu et al., 2018; Folkman, 2007). Our research found that ANGPT1-overexpressing N-DMSCs significantly enhance HMEC-1 viability, proliferation, migration, and tube formation capacity, and these enhancements can be reversed by Tie2 kinase inhibitors or angiogenesis inhibitors. In contrast, ANGPT1-knockdown P-DMSCs inhibit HMEC-1 viability, proliferation, migration, and tube formation capacity, and these inhibitory effects can be completely rescued by Tie2 activators. These results strongly confirm that the Ang1/Tie2 axis is a specific pathway through which DMSCs regulate HMEC-1 function in psoriasis. The selection of ANGPT1 as the focus of this study was based on data-driven screening. Among 15 angiogenesis-related genes examined, ANGPT1 and VCAM1 were significantly upregulated in P-DMSCs, while other factors such as FGF2, DLL4, and JAG1 showed no significant changes. Given its role as a secreted ligand that directly activates Tie2 receptors on endothelial cells, ANGPT1 represents a key paracrine signal from DMSCs to the vasculature. These findings, together with our previous observations of pro-angiogenic dysfunction in P-DMSCs (Li et al., 2018; Niu et al., 2016a; Niu et al., 2016b; Zhou et al., 2021) and previous reports of Ang1/Tie2 axis dysregulation in psoriatic lesions (Kuroda et al., 2001), provided the rationale for focusing on ANGPT1 in this study.

The signaling axis formed by the binding of Ang1 to the Tie2 receptor on ECs is a core mechanism for maintaining vascular stability and promoting vascular maturation under physiological conditions (Zhou et al., 2022; Zhang et al., 2024). However, in the chronic inflammatory environment of psoriasis, our findings suggest that Ang1/Tie2 signaling may play a “dual role” that is context-dependent. We hypothesize that DMSCs in psoriasis may be in a continuously “activated” state. Supporting this, we found that inflammatory cytokines, particularly TNF-α, directly induce ANGPT1 expression in DMSCs. These results indicate that infiltrating immune cells (e.g., Th17 cells, macrophages) release inflammatory cytokines that serve as key upstream signals driving Ang1 upregulation in P-DMSCs. The Ang1 secreted by these activated DMSCs may not only fail to induce vascular stability but also enhance the activated state of ECs. Our hypothesis is consistent with the recently proposed angiogenesis signaling network theory, which posits that crosstalk between different signaling pathways determines the final angiogenic phenotype (Song et al., 2025). Notably, our vascular maturity data showed that ANGPT1 overexpression increased vessel density but reduced pericyte coverage, accompanied by elevated Ang2 expression. These findings indicate that in the inflammatory microenvironment of psoriasis, ANGPT1 overexpression-induced neovessels lack adequate pericyte coverage and exhibit impaired maturation, further supporting the functional shift of Ang1 from a “pro-stabilizing” to a “pro-proliferative” mode. Future studies are needed to further clarify how Ang1/Tie2 signaling interacts with classical pathways in the psoriatic inflammatory microenvironment.

Furthermore, this study highlights the significance of DMSCs as regulators of angiogenesis. Under physiological conditions, DMSCs may maintain microvascular stability via paracrine signaling. However, in psoriasis, the function of disease-associated DMSCs undergoes significant changes, transforming from maintainers of vascular homeostasis to promoters of angiogenesis. This functional transformation may be associated with specific epigenetic modifications or continuous inflammatory signal stimulation in psoriatic lesions (Hou et al., 2017). Therefore, targeting DMSC function or intervening in their abnormal paracrine signals may represent a new strategy for psoriasis treatment. For example, the development of locally applied Tie2 receptor inhibitors or Ang1 neutralizers could specifically inhibit pathological angiogenesis without affecting systemic vascular stability.

To explore the potential mechanism of Ang1/Tie2-mediated interaction between DMSCs and ECs in psoriasis, we focused on the PI3K/AKT signaling pathway. Previous studies have confirmed that the PI3K/AKT pathway can promote angiogenesis and vascular remodeling and may coordinate the collective behavior of cells during vascular morphogenesis by regulating mechanical coupling between ECs (Li et al., 2025; Hu et al., 2021). It has also been reported that inhibiting Ang1 expression can inhibit the proliferation of psoriatic cells and that Ang1 can regulate the phosphorylation levels of PI3K and AKT in psoriatic cells (Rongna et al., 2018). Our results show that ANGPT1-overexpressing N-DMSCs significantly upregulate p-PI3K and p-AKT levels in HMEC-1. Meanwhile, PI3K inhibitors can completely reverse the promoting effects of ANGPT1 overexpression on HMEC-1 viability, proliferation, migration, and tube formation. In the co-culture model with ANGPT1 knockdown, the addition of a PI3K activator significantly reversed the shANGPT1-induced reduction in p-PI3K/p-AKT levels, and the functional inhibition of HMEC-1 (reduced viability, impaired proliferation, decreased migration, and reduced tube formation) was also restored. These findings indicate that the signals activated by Ang1 via Tie2 are transmitted downstream through the PI3K/AKT pathway to ultimately regulate EC function, filling the gap in our understanding of the mechanism underlying DMSC-EC interaction in psoriasis. In addition to the PI3K/AKT pathway, we found that the MAPK/ERK and STAT3 pathways were also activated downstream of Tie2 and partially contributed to Ang1-induced angiogenesis, albeit to a lesser extent. The coordinated activation of multiple downstream pathways may represent an important mechanism underlying excessive angiogenesis in psoriatic lesions and reflects the complexity of the angiogenic regulatory network.

Notably, Ang1 not only directly activates the Tie2 receptor to promote endothelial cell function but also upregulates VEGFA expression in endothelial cells via the PI3K/AKT pathway, forming a positive feedback amplification loop. This synergistic interaction between Ang1 and VEGF may represent an important mechanism underlying excessive vascular hyperplasia in psoriatic lesions.

Our *in vivo* experimental data strongly support the *in vitro* findings. In IMQ-induced mouse models, local regulation of Ang1 expression directly affected the severity of psoriasis-like lesions. Ang1 overexpression exacerbated lesion severity, epidermal hyperplasia, and local inflammation, while Ang1 knockdown effectively alleviated these pathological changes. This suggests that Ang1 may be involved in abnormal epidermal thickening either by directly regulating epidermal cell proliferation or indirectly via factors secreted by ECs, further linking the dual pathological features of vascular abnormalities and epidermal proliferation in psoriasis. As a core pathogenic factor in psoriasis, IL-17A can exacerbate the disease by inducing the release of inflammatory factors and promoting keratinocyte proliferation (Zhang L. et al., 2023). Our results showed that Ang1 overexpression not only enhances the expression of epidermal proliferation- and angiogenesis-related genes but also upregulates the expression of inflammatory factors such as IL-17A and TNF-α. Importantly, key findings were validated in primary HDMECs, confirming the generalizability and physiological relevance of our conclusions. Furthermore, we detected a mild upregulation of ANGPT2 mRNA in P-DMSCs and confirmed in mouse lesions that Ang2 expression is regulated by Ang1. Together with the report by Kuroda et al. showing elevated Ang2 expression in psoriatic lesions (Kuroda et al., 2001), our findings suggest that Ang1 and Ang2 may synergistically participate in the regulation of psoriasis-associated angiogenesis. These findings suggest that the Ang1/Tie2 axis may constitute an important bridge connecting the three core pathological links of “abnormal stromal cells - pathological angiogenesis - inflammatory infiltration” in psoriasis. Angiogenesis promoted by Ang1 provides channels and sites for inflammatory cell infiltration, and infiltrated inflammatory cells further release factors to maintain the activated state of DMSCs and high Ang1 secretion, forming a vicious cycle that collectively drives the chronic progression of the disease.

Of course, this study has some limitations. First, although we largely controlled for major heterogeneity through cell screening, age/gender matching, and standardized culture conditions, individual differences among DMSCs may still exist. Second, the *in vivo* regulation of Ang1 expression by local plasmid injection has limited efficiency; future studies should employ better delivery strategies. In addition, transcriptome or proteome sequencing was not performed in this study, and future omics analyses could provide a more comprehensive view.

**FIGURE 7:**
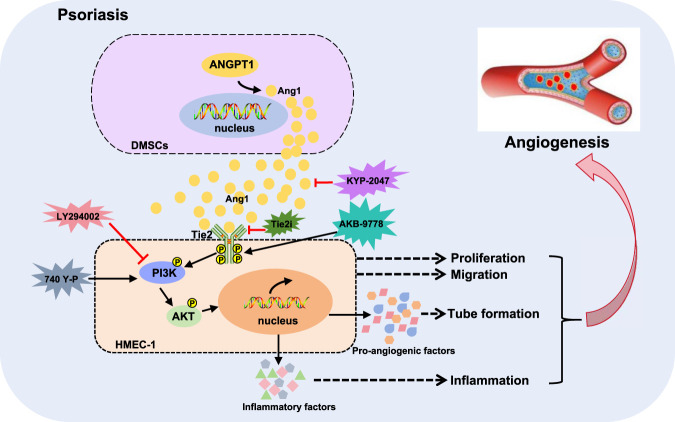
A schematic diagram illustrating the abnormal angiogenesis in the psoriasis lesion microenvironment mediated by the Ang1/Tie2 axis. In the DMSCs of psoriasis patients, the upregulated Ang1 binds to the Tie2 receptor on the surface of HMEC-1 through paracrine secretion, activating the downstream PI3K/AKT pathway, promoting the proliferation, migration and tube formation of HMEC-1, and ultimately leading to abnormal angiogenesis in the dermal papillary layer of psoriasis.

In conclusion, through *in vitro* cell experiments and *in vivo* animal models, this study confirms that abnormally high Ang1 expression in DMSCs from psoriatic patients is the key molecular basis for promoting angiogenesis. P-DMSCs secrete Ang1 via paracrine signaling, which binds to the Tie2 receptor on the surface of HMEC-1 and activates the downstream PI3K/AKT pathway, promoting HMEC-1 proliferation, migration, and tube formation, ultimately leading to the angiogenesis of psoriasis (Figure 7). Inhibiting Ang1 can alleviate these pathological changes. This research not only refines our understanding of the molecular mechanism of vascular abnormalities in psoriasis but also provides solid experimental evidence for the development of psoriasis treatment strategies targeting Ang1 or its signaling pathway.

## Data Availability

The raw data supporting the conclusions of this article will be made available by the authors, without undue reservation.

## Supplementary material

SUPPLEMENTARY FIGURE S1 Expression of Angiogenesis-related Genes in N-DMSCs and P-DMSCs. mRNA Expression levels of 15 angiogenesis-related genes in N-DMSCs and P-DMSCs were analyzed by qRT-PCR. All experiments were performed in triplicate (n = 6 per group). ns *p* > 0.05, **p* < 0.05.

SUPPLEMENTARY FIGURE S2 ANGPT1 Overexpression in N-DMSCs Promotes Angiogenesis in Primary HDMECs. **(A,B)** Percentage of EdU-positive primary HDMECs co-cultured with N-DMSCs transfected with pLVX-ANGPT1^high^ (scale bar = 100 μm). **(C,D)** Numbers of migrated HDMECs detected by Transwell assay (scale bar = 50 μm). **(E)** Phase-contrast micrographs of vascular-like structures formed by HDMECs (scale bar = 200 μm), and quantitative analysis of nodes **(F)**, meshes **(G)**, junctions **(H)**, and branches **(I)** of vascular-like structures. All experiments were performed in triplicate (n = 3 per group). ns *p* > 0.05, **p* < 0.05, ***p* < 0.01.

SUPPLEMENTARY FIGURE S3 ANGPT1 Knockdown in P-DMSCs Suppresses Angiogenesis in Primary HDMECs. **(A,B)** Percentage of EdU-positive primary HDMECs co-cultured with P-DMSCs transfected with pLKO.1-shANGPT1 (scale bar = 100 μm). **(C,D)** Numbers of migrated HDMECs detected by Transwell assay (scale bar = 50 μm). **(E)** Phase-contrast micrographs of vascular-like structures formed by HDMECs (scale bar = 200 μm), and quantitative analysis of nodes **(F)**, meshes **(G)**, junctions **(H)**, and branches **(I)** of vascular-like structures. All experiments were performed in triplicate (n = 3 per group). ns *p* > 0.05, **p* < 0.05.

SUPPLEMENTARY FIGURE S4 MAPK/ERK and STAT3 Pathways Are Activated Downstream of Tie2 and Partially Contribute to Ang1-Induced Angiogenesis. **(A–D)** Protein levels of p-ERK1/2 **(A,B)** and p-STAT3 **(C,D)** in HMEC-1 co-cultured with N-DMSCs transfected with pLVX-ANGPT1^high^ (with or without Tie2 inhibitor treatment) detected by Western blotting. **(E,F)** Percentage of EdU-positive HMEC-1 cells (scale bar = 100 μm). **(G,H)** Numbers of migrated HMEC-1 detected by Transwell assay (scale bar = 50 μm). **(I)** Phase-contrast micrographs of vascular-like structures formed by HMEC-1 (scale bar = 200 μm), and quantitative analysis of nodes **(J)**, meshes **(K)**, junctions **(L)**, and branches **(M)** of vascular-like structures. All experiments were performed with HMEC-1 co-cultured with ANGPT1-overexpressing N-DMSCs in the presence of PD98059 (10 μM) or Stattic (2 μM) as indicated. All protein levels normalized to β-actin. All experiments were performed in triplicate (n = 6 per group). ns *p* > 0.05, **p* < 0.05, ***p* < 0.01, ****p* < 0.001.

SUPPLEMENTARY FIGURE S5
*In Vivo* Validation of Ang1 Modulation and Ang2 Expression in Lesional Skin. **(A)** ANGPT1 mRNA levels in lesional skin of IMQ-induced mice treated with pLVX-Vehicle, pLVX-ANGPT1^high^, pLKO.1-shNC, or pLKO.1-shANGPT1 analyzed by qRT-PCR. **(B,C)** Ang1 protein levels in lesional skin detected by Western blotting. **(D)** Immunohistochemical staining of Ang2 in lesional skin (scale bar = 100 μm). **(E)** Quantification of Ang2-positive area. All protein levels normalized to β-actin. All experiments were performed in triplicate (n = 3 per group). ns *p* > 0.05, **p* < 0.05, ***p* < 0.01, ****p* < 0.001.

SUPPLEMENTARY FIGURE S6 Post-hoc power analysis using G*Power 3.1.9.2. The analysis was performed based on the ANGPT1 mRNA expression data (N-DMSCs: 1.252 ± 0.918, n = 18; P-DMSCs: 2.122 ± 0.840, n = 18). A two-tailed independent samples t-test was used with α = 0.05. The calculated Cohen’s d was 0.9888, and the achieved statistical power was 0.82.
