# Supplementary figures and images for: Dermal mesenchymal stem cells promote angiogenesis in HMEC-1 via activation of the angiopoietin 1/Tie2 pathway in psoriasis
Source: Front Cell Dev Biol. 2026 May 20;14:1771279. doi: 10.3389/fcell.2026.1771279 (PMC13230207; doi:10.3389/fcell.2026.1771279)

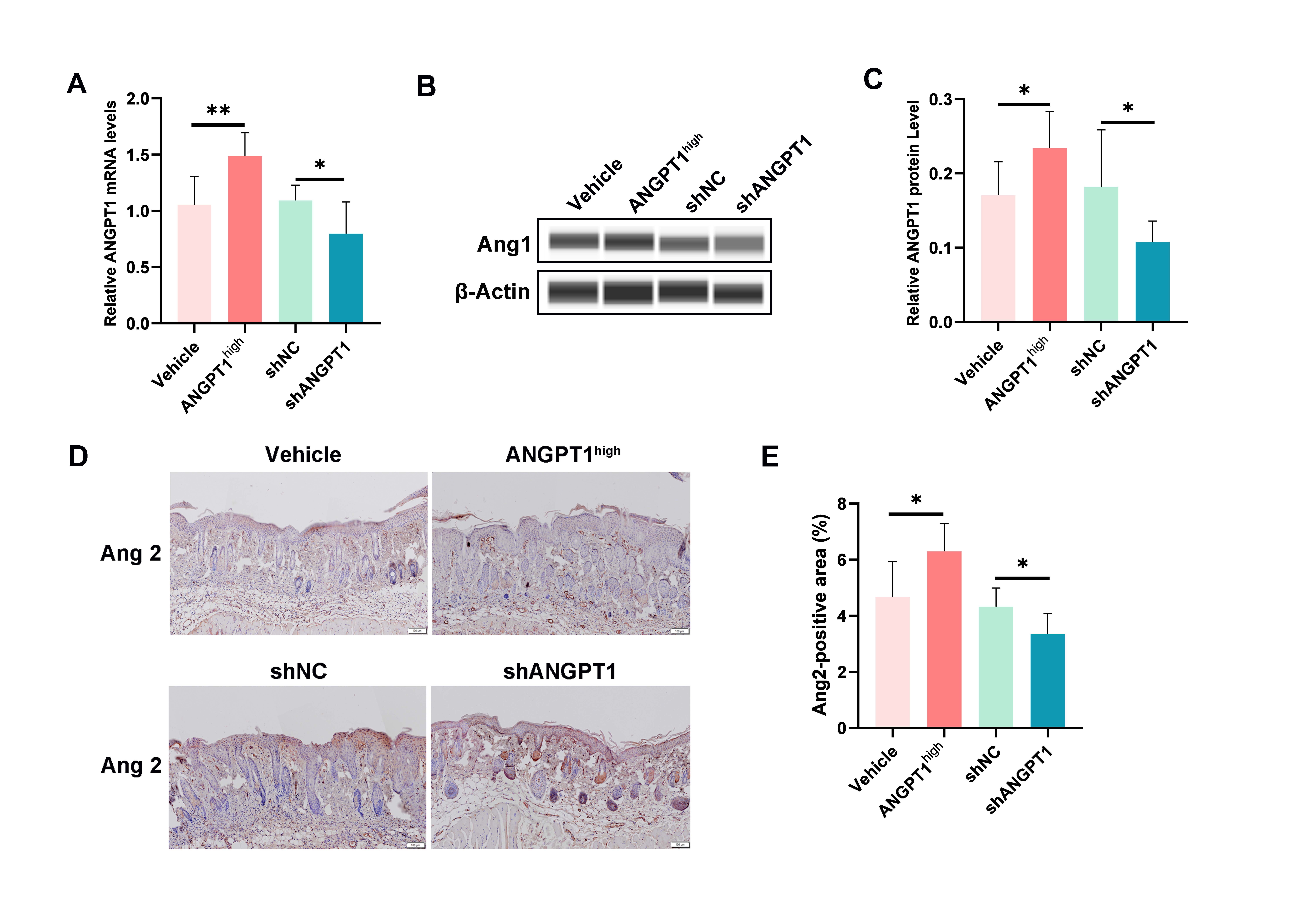

Supplement: Supplementary file 5 [file Image5.JPEG]

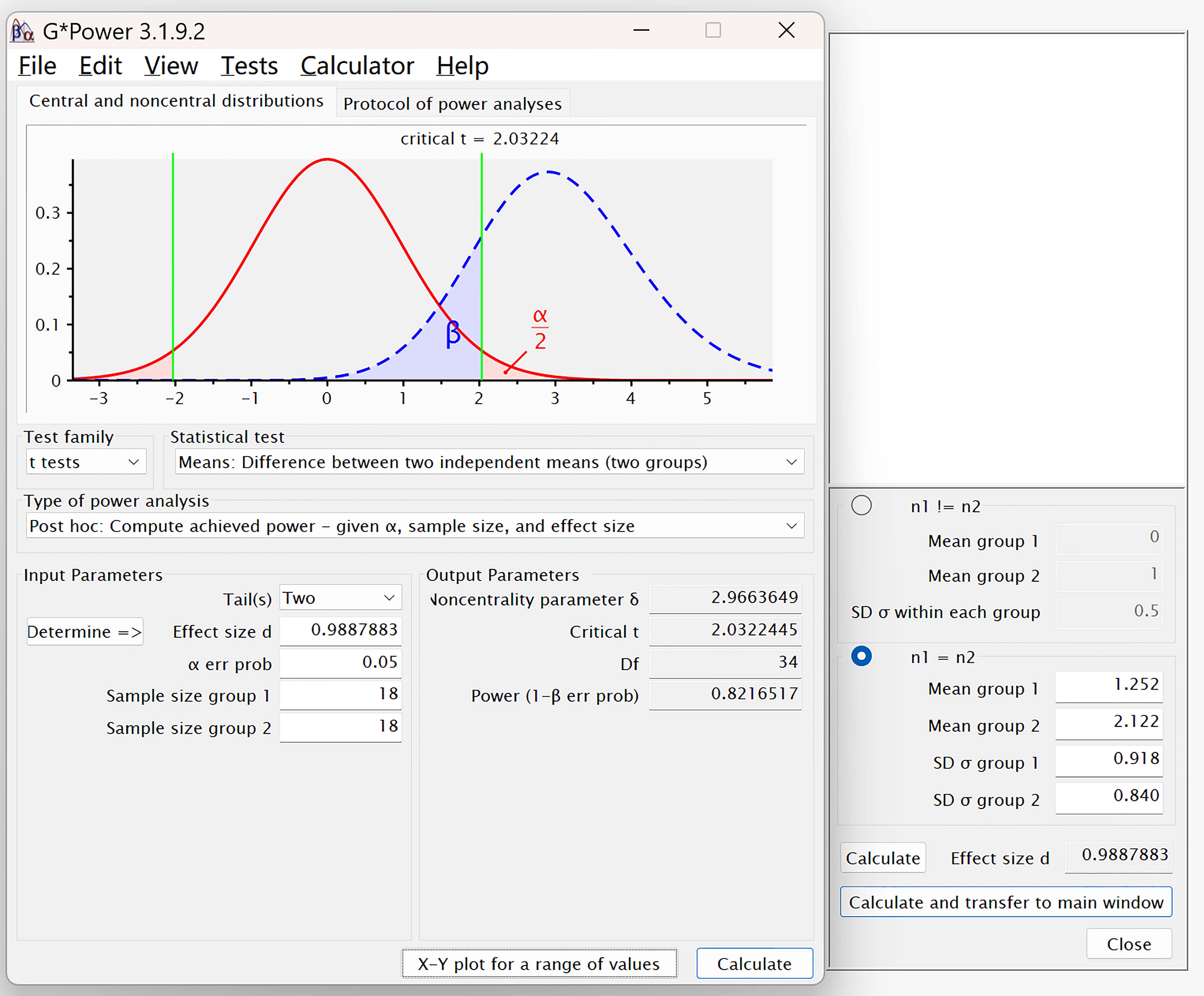

Supplement: Supplementary file 6 [file Image6.JPEG]
